# Supplementary material for: Determinants of COVID-19 vaccine uptake among persons with disabilities in three selected districts of Zambia
Source: PLOS Glob Public Health. 2025 Jul 7;5(7):e0003868. doi: 10.1371/journal.pgph.0003868 (PMC12233241; doi:10.1371/journal.pgph.0003868)
Supplement: S1 Table — (DOCX) [file pgph.0003868.s001.docx]

**The sample size Determination**

Sample size. The sample size was for Cross-sectional studies or cross-sectional survey using Cochrane´s formula (Charan and Biswas, 2013). Other studies have used the formula Mudenda et al., (2022). n=(Z^2 p(1-p))/d^2

Table 1: Sample size estimated

| **Description of the Parameter** | **Value** |
| --- | --- |
| d = absolute precision | 0.05 |
| p = average expected proportion of disease in the population | 0.7 |
| Z(1-α⁄2) = value of the standard distribution corresponding to α | 1.96 |
| The probability of erroneously rejecting the null hypothesis when it was correct (α) was set at | 5% (Z = 1.96). |
| n=((1.96)^2 (0.7)(1-0.7))/〖(0.05)〗^2 | |
| n = (3.8416×0.7×0.3)/0.0025 | 322.69 |
| Sample size | 323 |
| The non-response rate estimate | 20% |
| Accounting for design effect | 1.5 |
| Final sample size determined | 549 |

The final sample obtained was 985. The sample was allocated to the selected districts using the proportional to size technique as indicated below.

The total proportion estimated at 985 of registered PWDs on the Social welfare register (receiving social cash transfer and those yet to start receiving) Lusaka= 55% (5430),

Mazabuka 21% (2055) and Monze 24% (2370).

Based on the total estimates the final sample size for each district was as follows:

**Table 2: Proportion to the District of study**

| **District** | **Proportional to Size sample** |
| --- | --- |
| Lusaka District final sample size 985 (55)/100 | 543 |
| Mazabuka District sample size 985 (20.6)/100 | 205 |
| Monze District sample size 985 (24)/100 | 237 |
