# Supplementary material for: Determinants of COVID-19 vaccine uptake among persons with disabilities in three selected districts of Zambia
Source: PLOS Glob Public Health. 2025 Jul 7;5(7):e0003868. doi: 10.1371/journal.pgph.0003868 (PMC12233241; doi:10.1371/journal.pgph.0003868)
Supplement: S2 Table — (DOCX) [file pgph.0003868.s002.docx]

**S2 Table:** **Self-Reported Determinants of the Health Belief Model Consonants and COVID-19 Uptake among PWDs**

| **Health Belief Model Consonants** | Vaccinated  n=666 (67.61%) | Un-vaccinated n= 319 (32.39%) | Total N=985 | p-value |
| --- | --- | --- | --- | --- |
| **Perceived Susceptibility** |  |  |  |  |
| Have you ever heard about COVID-19 Disease? |  |  |  |  |
| No | 7 (1.05) | 12 (3.76) | 19 (1.93) | 0.004^c^ |
| Yes | 659 (98.95) | 307 (96.24) | 966 (98.06) |  |
| COVID-19 may be a risk to you. What do you think applies in your case? (Likely) |  |  |  |  |
| No Risk | 186 (27.93) | 135 (42.32) | 321 (32.59) |  |
| Minor Risk | 83 (12.46) | 44 (13.79) | 127 (12.89) |  |
| Not sure | 46 (6.94) | 31 (9.72) | 77 (7.82) | <0.001^c^ |
| Moderate Risk | 49 (7.36) | 25 (7.84) | 74 (7.51) |  |
| High Risk | 302 (45.35) | 84 (26.33) | 386 (39.19) |  |
| COVID-19 a Risk to members of the Public |  |  |  |  |
| No Risk | 348 (52.25) | 147 (46.08) | 495 (60.23) |  |
| Minor Risk | 84 (12.61) | 45 (14.11) | 129 (13.10) |  |
| Not Sure | 38 (5.71) | 35 (10.97) | 73 (7.41) | 0.006^c^ |
| Moderate Risk | 31 (4.65) | 24 (7.52) | 55 (5.58) |  |
| High Risk | 165 (24.77) | 68 (21.32) | 233 (23.65 ) |  |
| **Perceived Severity** |  |  |  |  |
| How likely do you think you might be infected and be hospitalised with Covid-19 in the future? (Likely) |  |  |  |  |
| Not Likely | 231 (34.74) | 157 (49.22) | 388 (39.43) |  |
| Somewhat likely | 129 (19.40) | 61 (19.12) | 190 (19.31) | <0.001^c^ |
| Likely | 120 (18.05) | 34 (10.66) | 144 (15.65) |  |
| Most Likely | 185 (27.82) | 67 (21.00) | 252 (25.61) |  |
| Have you ever heard of or seen a person with signs and symptoms of COVID-19 or died of COVID-19? (Yes) |  |  |  |  |
| No | 395 (59.31) | 210 (66.25) | 605 (61.55) | 0.037^c^ |
| Yes | 271 (40.69) | 107 (33.75) | 378 (38.45) |  |
| Have you been infected with COVID-19 before? |  |  |  |  |
| No | 642 (96.40) | 316 (99.06) | 958 (97.26) |  |
| Yes | 24 (3.60) | 30 (0.94) | 27 (2.74) | 0.017^c^ |
| **Perceived Benefits** |  |  |  |  |
| Reasons Vaccination Status |  |  |  |  |
| The Safety of the COVID-19 Vaccine |  |  |  |  |
| No | 15 (2.25) | 59 (18.61) | 74 (7.57) | <0.001^c^ |
| Yes | 651 (97.75) | 258 (81.39) | 909 (92.42) |  |
| The effectiveness of COVID-19 vaccine |  |  |  |  |
| No | 7 (1.05 | 32 (10.09) | 39 (3.98) |  |
| Yes | 621 (93.52) | 249 (78.55) | 670 (88.69) | <0.001^c^ |
| Not sure | 36 (5.42) | 36 (11.36) | 72 (7.34) |  |
| COVID-19 vaccine meant to Protect |  |  |  |  |
| Agree | 655 (98.94) | 282(95.27) () | 937 (97.81) | <0.001^c^ |
| Disagree | 7 (1.06) | 14 (4.73) | 21 (2.19) |  |
| **Perceived Barriers** |  |  |  |  |
| **Ever been vaccinated** |  |  |  |  |
| No | 137 (20.57) | 140 (43.89) | 277(28.12) | <0.001^c^ |
| Yes | 529 (79.43) | 179 (56.11) | 708 (71.88) |  |
| **Cause fertility complications in people** |  |  |  |  |
| Strongly agree | 4 (0.60) | 3 (0.96) | 7 (0.72) |  |
| Agree | 6 (0.91) | 5 (1.59) | 11 (1.13) |  |
| Not sure | 188 (28.40) | 85 (27.07) | 273 (27.97) | 0.009^f^ |
| Disagree | 320 (48.34) | 179 (57.01) | 499 (51.13) |  |
| Strongly Disagree | 144 (21.75) | 42 (13.38) | 186 (19.06) |  |
| **COVID-19 vaccine causes illness and dearth** |  |  |  |  |
| Strongly agree | 11 (1.65) | 4 (1.27) | 15 (1.53) |  |
| Agree | 41 (6.17) | 35 (11.11) | 76 (7.76) |  |
| Not sure | 136 (20.45) | 76 (24.13) | 212 (21.63) | 0.002^f^ |
| Disagree | 373 (56.09) | 173 (54.92) | 546 (55.71) |  |
| Strongly Disagree | 104 (15.64) | 27 (8.57) | 131 (13.37) |  |
| **COVID-19 Human Caused disease** |  |  |  |  |
| Strongly Agree | 22 (3.31) | 16 (5.08) | 38 (3.88) |  |
| Agree | 100 (15.04) | 57 (18.10) | 157 (16.20) |  |
| Not sure | 208 (31.28) | 101 (32.06) | 309 (31.33) | 0.215^c^ |
| Disagree | 239 (35.94) | 108 (34.29) | 347 (35.41) |  |
| Strongly disagree | 96 (14.44) | 33 (10.48) | 129 (13.16) |  |
| **Social Support** |  |  |  |  |
| Yes | 366 (54.95) | 140 (43.89) | 506 (51.37) | 0.001^c^ |
| No | 300 (45.05) | 179 (56.11) | 479 (48.63) |  |
| **Amount Received from Social Support** |  |  |  |  |
| K200 | 112 (30.77) | 25 (17.99) | 137 (27.34) |  |
| K400 | 236 (64.84) | 105 (75.54) | 341 (67.79) | 0.013^c^ |
| K800 | 16 (4.40) | 9 (6.47) | 25 (4.97) |  |
| **Other income earned** |  |  |  |  |
| < k200 | 634 (95.20) | 312 (97.81) | 946 (96.04 | 0.049^f^ |
| >K 200 | 32 (4.80) | 7 (17.954) | 39 (3.96) |  |
| **Capacity to Visit Health Facility** |  |  |  |  |
| Yes | 329 (49.40) | 132 (41.38) | 461 (46.80) | 0.018^c^ |
| No | 337 (50.60) | 187 (58.62) | 524 (53.20) |  |
| **Health Education on COVID-19** |  |  |  |  |
| Yes | 608 (91.43) | 264 (84.62) | 872 (89.25) | 0.001^c^ |
| No | 57 (8.57) | 48 (15.38) | 105 (10.75) |  |
| Visited health facility for routine health checks |  |  |  |  |
| No | 131 (24.30) | 188 (19.09) | 319 (32.39) | <0.001^c^ |
| Yes | 408 (75.70) | 258 (57.85) | 666 (67.61) |  |
|  |  |  |  |  |
|  |  |  |  |  |
| **Other Consonants with proportions non-vaccinated** | Yes | No |  |  |
| Fear of an injection | 79 (26.87) | 268 (76.44) | 100 (294) |  |
| I can recover easily without a vaccine | 7 (2.38) | 287 (97) | 100 (294) |  |
| My Immune system is very strong against COVID-19 vaccine | 4 (1.36) | 290 (98.9) | 100 (294) |  |
| I have not decided about a vaccine uptake (Wait and see) | 46 (15.65) | 279 (95) | 294 (100) |  |
| Don’t trust the vaccine | 25 (8.50) | 269 (91.49) | 294 (100) |  |
| Don’t trust the pharmaceutical industries | 0.68 (2) | 292 (99) | 294 (100) |  |
| I have no information about vaccination | 12 (4.08) | 282 (95.90) | 294 (100) |  |
| I don’t get vaccinated due to religious reasons | 4 (1.36) | 290 (98.60) | 294 (100) |  |
| From history, I don’t get vaccinated | 7 (2.38) | 287 (97.60) | 294 (100) |  |
| My health status doesn’t allow me to be vaccinated | 43 (14.63) | 279 (85) | 313 (100) |  |
| **Ques to Action** |  |  |  |  |
| Information on the existence of COVID-19 |  |  |  |  |
| Strongly agree | 187 (28.08) | 61 (19.24) | 248 (25.23) |  |
| Agree | 409 (61.41) | 200 (63.09) | 609 (61.95) |  |
| Not sure | 47 (7.06) | 33 (10.41) | 80 (8.14) | 0.002^c^ |
| Disagree | 14 (2.10) | 11 (3.47) | 25 (2.54) |  |
| Strongly Disagree | 9 (1.35) | 12 (3.79) | 21 (2.14) |  |
| Proportions on the Ques to Action |  |  |  |  |
| Protect me against covid-19 | 464 (69.67) | 262 (30.33) | 666 (100) |  |
| Told by a government worker | **31 (4.65)** | 635 (95.34) | 666 (100) |  |
| Protect others from COVID-19 | 74 (11.11) | 592 (88.3) | 666 (100) |  |
| Fear of being Infected of COVID-19 | 49 (7.36) | 617 (92.64) | 666 (100) |  |
| Recommendation by parents | 11 (1.65) | 655 (98.34) | 666 (100) |  |
| Get read of the Virus | 6 (0.90) | 660 (99.09) | 666 (100) |  |

^c^Chi-square, ^f^Fishers Exact Test, **^c^Chi-square test, ^f^Fishers Exact Test, ^e^Exchange rate ZMW 22= USD 1.**
