# Supplementary material for: Determinants of COVID-19 vaccine uptake among persons with disabilities in three selected districts of Zambia
Source: PLOS Glob Public Health. 2025 Jul 7;5(7):e0003868. doi: 10.1371/journal.pgph.0003868 (PMC12233241; doi:10.1371/journal.pgph.0003868)
Supplement: S1 Text — (PDF) [file pgph.0003868.s003.pdf]

THE UNIVERSITY OF ZAMBIA

GRADUATE SCHOOL OF PUBLIC HEALTH

LUSAKA-ZAMBIA

Barriers and facilitators (Determinants) in Accessing Primary Health Care and COVID-19 Vaccines During and after the COVID-19 pandemic among people living with disabilities in Zambia.

Introduction for Respondent

My name is **Allan Mayaba Mwiinde**, a postgraduate student at the University of Zambia (UNZA), conducting Research on Barriers and facilitators (Determinants) in Accessing Primary Health Care and COVID-19 Vaccines During and after the COVID-19 pandemic among people living with disabilities in Zambia. The purpose of this study is to enable me, in part, to fulfill the requirements for the award of Doctor of Philosophy in Epidemiology. As a citizen/parent/Guardian of Lusaka, Monze or Mazabuka District of age above 18 years, you have been selected as a respondent in this study and I would like to ask you some questions. The information you give will be kept strictly confidential. Therefore, I encourage you to be as open and free as possible.

1. General Respondent Information

Date of Interview:			
Name of Interviewer:			
Name of Respondents:			
Sex of Respondent	Male	Female	Age
District of Residence			
Area of Residence			
Village of Residence/Chief			
Religion			
Denomination			
Settlement			
Occupation			

Estimated Monthly Income					
Which sector do you belong to	Informal Sector		Formal Sector	Unemployed	
Marital Status	Single	Married	Divorced	Widowed	Single Parent
Level of Education	Never went to School	Primary	Basic Level	Secondary	Tertiary
Qualification Obtained	Crafts Certificate	Workshop Attendance	Diploma	Degree	Postgraduate

Occupation Details

1. What is your occupation.?
    a. Commercial Farmer
    b. Peasant Farmer
    c. Farm Worker
    d. Fisherman
    e. Trader please specify commodity………………………………..
    f. Civil Servant Please State the occupation ……………………………..
    g. Any other please state…………………………………………….

Additional data capture for People Living with Disabilities

**Type of Disability**	**Please Tick**	**Congenital Disability**	**Acquired Disability**
**Mobility/Physical impairment**			
**Spinal cord disability**			
**Head injuries-Brain disabilities**			
**Vision Disability**			
**Hearing Disability**			

**Cognitive or learning disabilities**			
**Psychological disorders**			
**Invisible Disabilities**			
**Any other disabilities not mentioned above**			

Vaccination Information

1. Have you ever heard about COVID-19 Disease? Yes ( ) No ( )
2. Have you ever been infected with COVID-19? Yes ( ) No ( )
3. How likely do you think you might be infected with Covid-19 in the future?
   a. Not Likely
   b. Somewhat likely
   c. Likely
   d. Most Likely

4. Does COVID-19 cause a risk to you
   a. No risk
   b. Minor risk
   c. Moderate risk
   d. High risk
   e. Not sure
5. Does COVID-19 cause a risk to members of the Public
   a. No risk
   b. Minor risk
   c. Moderate risk
   d. High risk
   e. Not sure

6. Have you ever heard of or seen a person with signs and symptoms of COVID-19? Yes ( ) No ( )
7. Have you been infected with COVID-19 during the pandemic Yes ( ) No ( ).
8. If yes to the above question which period of the COVID-19 wave?
   a. First wave
   b. Second Wave
   c. Third Wave
   d. Fourth Wave

9. Do you think that vaccines are safe in general Yes ( ) No ( ) Not sure ( )
10. Do you think vaccines are effective in general Yes ( ) No ( ) Not sure ( )
11. Have you ever been vaccinated in the past Yes ( ) No ( )
12. Have you ever heard about COVID-19 Vaccines? Yes ( ) No ( )
13. If yes, to question 6 when did you hear about the Vaccine?
    a. In 2020
    b. In 2021
    c. In 2022
    d. In 2023
    e. If possible, the month…….
14. If yes, what have you heard about the COVID-19 vaccine?
    a. It is meant to protect against the COVID-19 virus
    b. It is targeting at killing people who will get vaccinated
    c. It is there to cause COVID-19 disease in people
    d. It is there for unknown reasons
    e. Any other please state…………………………………….
15. Have you heard any of a conspiracy against vaccines? (Yes) (No)
16. If yes to the question above, how the conspiracy affected you
    a. Made you develop a negative attitude toward vaccines
    b. Made you develop a positive attitude about the vaccine
    c. I don't know
17. Have you been vaccinated? Yes ( ) No ( )?
18. If yes which vaccine product?
    a. Pfizer
    b. Moderna
    c. Jonson and Jonson
    d. AstraZeneca
    e. Covishield
    f. Sinopharm
19. If yes what stage of vaccination?
    a. Single dose only
    b. Full dose only
    c. Booster vaccine
20. If yes, where were you vaccinated from? …………………………………
21. If yes what are the reasons for your vaccination?
    a. I was infected by COVID-19
    b. To protect me against COVID-19
    c. To protect others from COVID-19
    d. To be able to travel

e. It is a social and moral responsibility
f. I was told by the government worker
g. It is mandatory to get vaccinated in our organization
h. I saw my friend/Relative being vaccinated
i. For fear of getting infected with COVID-19
j. Parent recommendation
k. To get read of the virus and end the pandemic
l. Any other please state the reason………………………………….

22. If yes, have you also taken the COVID-19 booster vaccine? Yes ( ) No ( )
23. If yes, why did you take the booster vaccine?
    a. To further protect myself from COVID-19
    b. To protect myself from the new variant of COVID-19
    c. To protect others from COVID-19
    d. I was just told to take the booster vaccine
    e. Any other please state……………………………………….
24. If vaccinated but not taken the booster vaccine, why?
    a. The booster vaccine is not available
    b. I don't need to take the booster vaccine I am already immune
    c. I don't like the pain of taking a vaccine
    d. The first and second dose of the vaccine is enough to protect my immunity
    e. The booster vaccine is not safe for the health
    f. Any other please state………………………………………………….
25. If yes, you are vaccinated what were the vaccine signs and symptoms you developed after intake for the first time?
    a. Mild Headache
    b. Severe Headache
    c. Fever
    d. Cough
    e. Flu
    f. Pain on the side of the injection
26. If yes, have you ever had a severe allergic reaction (e.g., anaphylaxis) in the past? Example: a reaction for which you were treated with epinephrine or EpiPen, or for which you had to go to the hospital? Yes ( ) No ( )
27. If yes, how was the reaction?
    a. severe allergic reaction after receiving a COVID-19 vaccine
    b. severe allergic reaction after receiving a second dose of COVID-19 vaccine.
    c. Mild reaction after receiving the COVID-19 booster vaccine
28. If yes vaccinated, do you trust the COVID-19 vaccine? Yes ( ) No ( )
29. If vaccinated, would you recommend a friend or family member to get the COVID-19 vaccine

a. Yes ( )
b. No ( )

30. If yes how many of your family members are vaccinated? …………………
31. If not vaccinated why?
    a. Fear of an injection
    b. I am not sure if the COVID-19 vaccine effective
    c. I am not sure of COVID-19 vaccine safety
    d. I want to wait until more people take it
    e. I can recover easily without a vaccine
    f. I have not decided about a vaccine uptake
    g. Fear of getting sick of COVID-19
    h. Don't trust the vaccine
    i. Don't trust the government
    j. The vaccine is not available
    k. I have no information about vaccination
    l. I don't get vaccinated due to religious reasons
    m. From history I don't get vaccinated
    n. Any other reason please state……………………………………
32. If no, to question NOT vaccinated what is the likely hood of you being vaccinated?
    a. Most likely
    b. Not Likely
    c. Somewhat likely

Now I am going to ask you questions about access to Primary Health Care for People Living with Disabilities

Primary Health Care Questionnaire

1. Whom do you stay with for social support
    a. Alone
    b. Wife/Husband
    c. Daughter/Son
    d. Father/Mother
    e. Family member
    f. Friend
    g. Well-wisher
    h. Any other please state…………………
2. Do you have a Primary Health Care facility within your ward? Yes ( ) No ( )
3. If yes how far is it from your house approximately
    a. Less than 1km
    b. Less than 5km
    c. Less than 10km
    d. Less than 15 km
    e. Any other please specify …………………..
4. What mode of transport do you use to reach the health facility

a. Walking on foot
b. Bicycle/Motorcycle
c. Motor vehicle
d. Oxcart
e. Any other please mention ……………………………….

5. Is the transport system you have indicated affordable Yes ( ) No ( ).
6. Approximately how many minutes/hours does it take to reach the Health Facility
   a. Less than 30 minutes
   b. Less than 1-hour
   c. Less than 2 hours
   d. Less than 3 hours
   e. Less than 4 hours
7. Is the road easily accessible to the health facility Yes ( ) No ( ).
8. If yes is it accessible during the rainy season Yes ( ) No ( )
9. What is the state of the road network system to reach the Primary Health Care Facility
   a. Poor Gravel Road
   b. Fair Gravel Road
   c. Good Gravel Road
   d. Tared Bituminous Road
   e. Pedestrian path road
   f. Any other please specify …………………
10. Do you have a health condition that requires you to have access to Primary Health Care time and again? Yes ( ) No ( ).
11. If no to the above question what motivates you from seeking Primary Health care services
    a. When I don't feel well
    b. When I just want to have a routine checkup
    c. When It has been a long I ever went to the facility
    d. When I just want to consult a health worker
    e. Any other please indicate ……………………………………………….
12. If yes to question 9 how often do you visit Primary Health Care facilities for the mentioned condition?
    a. Never
    b. Rarely
    c. Sometimes
    d. Always
13. If no to question 7, did you visit the health care facility during the COVID-19 pandemic Yes ( ) No ( ).
14. If yes were the Primary Health Care staff accommodative?
    a. Not accommodative
    b. Slightly accommodative
    c. Moderately accommodative
    d. Highly accommodative
15. Do you think healthcare workers are adequately skilled for their job? Yes ( ) No ( ).
16. If No to the question above which area need attention
    a. Disease Screening section

b. Disease detection (Laboratory) section
c. Nursing Section
d. Community health section
e. Health education section
f. Any other please state…………………………………………………

17. Did the health care workers meet your expectations Yes ( ) No ( ).
18. If no what do you think they did not do well
    a. The healthcare workers were too few
    b. The health care workers were too busy
    c. The health care workers were not active on duty
    d. The healthcare workers did not pay attention to my health problem
    e. Any other please mention ……………………………………………….
19. If the health care did not meet your expectation, what did you do thereafter
    a. Wait for another period of a hospital visit
    b. Provided self-medication
    c. Provided with self-traditional medicines
    d. Went for traditional healers
    e. Went back to a health facility
    f. Any other please specify ……………….
20. Following your experience at the health center do you trust the health care services being offered
    a. I don't trust
    b. I somehow trust
    c. I trust
    d. I fully trust
21. Did the Health facility have the medication available
    a. Not available
    b. Some available
    c. Available
    d. Adequately available
22. Have you ever been told to go and buy your own medicine Yes ( ) No ( ).
23. If yes to question 22 how likely are you told to buy medicines
    a. Very unlikely
    b. Unlikely
    c. Likely
    d. Extremely Likely
24. If yes what is the cost of medication in Zambian Kwacha…………………………..
25. Are the health services affordable at the health facilities Yes ( ) No ( ).
26. If no what is do you think is not affordable
    a. The cost of medicines
    b. The cost of consultation fees
    c. The cost of food at the health facility
    d. The cost of admission at the health facility
27. Are you on any Health Scheme/Insurance to help finance your health bills Yes ( ) No ( ).
28. If yes, please name the Health Scheme provider ……………………….
29. If not, how do you finance your health service bills

a. Personal payments
b. Family members contribute
c. Organization pays the bills
d. Well-wishers pay the bills
e. Any other please indicate …………….

30. If not what is the cost of the medical bills approximately …………………………..
31. Do the health facility provide you with information to help you manage any health condition you have reported. Yes ( ) No ( ).
32. What do you think can be improved to better Please state …………………………..

Thank you for your time and responding to the Questionnaire

End of Questionnaire.
