# Supplementary material for: Determinants of COVID-19 vaccine uptake among persons with disabilities in three selected districts of Zambia
Source: PLOS Glob Public Health. 2025 Jul 7;5(7):e0003868. doi: 10.1371/journal.pgph.0003868 (PMC12233241; doi:10.1371/journal.pgph.0003868)
Supplement: Attachment — Submitted filename: PLOS review.docx [file pgph.0003868.s005.docx]

Comments:

Abstract: Abstract should be revised to a more concise format. The first two lines of the abstract do not contribute significantly to the paper.

Revise ‘determinants of vaccine uptake’ to ‘odds of vaccine uptake’

‘Safety of the vaccine’- statement is incomplete.

Introduction:

Line 52-59: These should be revised, as the paper focuses on uptake, with the assumption that vaccines are already available in the country studied, not on vaccine apartheid which is not relevant to the paper.

Lines 60-65: These should be moved from introduction to discussion.

Lines 71-73: Same comments as lines 60-65

There are other studies that have investigated the uptake of COVID-19 vaccines among other populations in Zambia and should be included to provide a better summary of the COVID-19 vaccine situation in Zambia. The introduction should be revised, and be written in amore concise manner, with a direct focus on the topic and outcomes of interest.

Methods:

Lines 120- 122: Define low uptake- There is either uptake or no uptake, not low uptake. Revisit the SAGE definition, as the definition provided is for vaccine hesitancy. It is not correct to assume that all participants who had not yet received a COVID-19 vaccine were hesitant, as the participants are a vulnerable group who would have significant limitations in accessing vaccine services.

Lines 126-128: It appears that the authors are conflating non-uptake, with willingness to receive a COVID-19 vaccine (unsure/undecided). It appears that there are two outcomes being investigated- uptake among the study population, and willingness to receive among those who had not yet received a COVID-19 vaccine. The authors should separate the outcomes, and treat them as two different outcomes.

Line 133: ‘participants randomly selected’. Insert ‘were’

Line 210: Same comments as in lines 126-128

Results:

Line 223: Include the proportion of male/female participants in the first lines of the results.

Table 1: Include the number and proportion of vaccinated and unvaccinated in Table 1. The sum of the cell percentages should add up to 100% based on either the row total or the column total for that variable, and not the total number of participants. E.g proportion of vaccinated participants aged 18-35 should either be 230/666 (number vaccinated as denominator- (34.5%)), or 230/375 (denominator is total participants aged 18-35 (61.3%)). Authors should do the same for all cells.

Table 1: Income should either be reported in ZMW or USD, and be consistent in the result narrative and the table.

Fig 3: The text speaks to ‘Type of disability and the proportion of COVID-19 vaccine uptake’, however the title of Fig 3 reads ‘The proportion of Disability by sex among PWDs’. Please revise.

Line 259: Past behavior towards vaccination- data not included in table 1.

Line 272: Change ‘servility’ to severity.

Line 307: This should be table 2, table 4.

Table 2:

Is the dependent variable vaccine uptake or vaccine acceptance? A person may accept a vaccine but have not yet actually received the vaccine (uptake).

Row not aligned

Why were variables not significant at 5% level of significance included in the unadjusted logistic regression model included in the adjusted model (e.g extent of disability, vision, hearing, speech, infected with COVID-19 before)? I suggest they are removed from the unadjusted model.

Discussion:

Line 346: What are the determinants of COVID vaccine uptake in the general population, and how do they vary from those in the study?

Line 347: There are no results for vaccine acceptance. The authors should be consistent with vaccine uptake.
