# Supplementary material for: Determinants of COVID-19 vaccine uptake among persons with disabilities in three selected districts of Zambia
Source: PLOS Glob Public Health. 2025 Jul 7;5(7):e0003868. doi: 10.1371/journal.pgph.0003868 (PMC12233241; doi:10.1371/journal.pgph.0003868)
Supplement: Attachment — Submitted filename: PLOS One_Vaccine study.doc [file pgph.0003868.s007.doc]

**Abstract**

The first sentence is contradictory to both parts of it.

A couple of typos/grammar issues

**Introduction**

line 43-46: The sentence needs to be rephrased into two sentences.

The second paragraph is not connected to the first one. There should be a connecting sentence in the first linking it to the second one.

Line 50: Since and currently mean the same thing. Expunge one.

Line 59: First mention of WHO, and it needs to be in full.

Line 66: The assertion made here needs to be revised as there a couple of articles on the subject matter. 1. <https://pmc.ncbi.nlm.nih.gov/articles/PMC9209920/> 2. <https://bmcpublichealth.biomedcentral.com/articles/10.1186/s12889-023-15116-w> 3. <https://pmc.ncbi.nlm.nih.gov/articles/PMC9159554/>

The study has failed to mention or bring out its importance in the introduction. For a study like this, lesson learned will guide future vaccine policies and efforts. Vaccine hesitancy was high in Africa for the Covid-19 and other vaccines in the past like the polio vaccine. So, what will the study contribute to knowledge?

Also, the introduction has too many paragraphs. For instance lines 88 and 89, a paragraph?

**Methodology**

Line 100: Spacing issues

Why the three districts ? What informed this decision ? Why just three months ?

Line 149: Why was the 50% assumption used? A prevalence rate study from another country from Africa would suffice?

The final sample size not clear at all. Why not present the sample size calculations using a Table and sent to the supplementary file.

Lines 164-170: Different font size and style.

Line 164: Was it open or closed ended ?

The disabilities of the respondents is not described/ What proportion were classified as physical, mental, etc ?

Line 201: What variables formed the dependent and independent variables? This is not clear.

Line 211: Why is the “P” in public in capital?

Line 198: Was it Chi square or Fisher exact that was applied in Table 1? if yes, the statement here has to be rephrased to reflect what was analysed. The result indicates vaccinated and un-vaccinated

Table 1 has got superscripts but there is legend to explain these.

Better graphics can be made from origin or even R for this bar charts (Figure 1 and 2).

Line 311: What does Ques means ?

**Results**

Numerous typos here.

**Limitation**

It is obvious that the authors adopted a closed ended questions. This should be mentioned in the limitations of the study. Using open ended questions would have led to the development of themes to guide the discussions better.
